# Developing rights-based standards for children having tests, treatments, examinations and interventions: using a collaborative, multi-phased, multi-method and multi-stakeholder approach to build consensus

**DOI:** 10.1007/s00431-023-05131-9

**Published:** 2023-08-11

**Authors:** Lucy Bray, Bernie Carter, Joann Kiernan, Ed Horowicz, Katie Dixon, James Ridley, Carol Robinson, Anna Simmons, Jennie Craske, Stephanie Sinha, Liza Morton, Begonya Nafria, Maria Forsner, Anna-Clara Rullander, Stefan Nilsson, Laura Darcy, Katarina Karlsson, Cath Hubbuck, Maria Brenner, Sian Spencer-Little, Kath Evans, Andrew Rowland, Carol Hilliard, Jennifer Preston, Piet L. Leroy, Damian Roland, Lisa Booth, Jean Davies, Holly Saron, Marie Edwinson Mansson, Ann Cox, Karen Ford, Steven Campbell, Julie Blamires, Annette Dickinson, Michael Neufeld, Blake Peck, Marla de Avila, Veronica Feeg, Henny Suzana Mediani, Maha Atout, Maureen D. Majamanda, Natasha North, Christine Chambers, Fanny Robichaud

**Affiliations:** 1 Edge Hill University, Ormskirk, UK; 2 Edge Hill University and Alder Hey Children’s Hospital, Liverpool, UK; 3 University of Liverpool, Liverpool, UK; 4 Expert by Experience, Liverpool, UK; 5 Edge Hill University and National Restraint Reduction Network, Ormskirk, UK; 6 University of Strathclyde, Glasglow, UK; 7 Alder Hey Children’s Hospital, Liverpool, UK; 8 University of Glasgow, Glasgow, UK; 9 Sant Joan de Déu Children’s Hospital, Barcelona, Spain; 10 Umeå University, Umeå, Sweden; 11 University of Gothenburg, Gothenburg, Sweden; 12 University of Borås, Borås, Sweden; 13 Great Ormond Street Hospital, London, UK; 14 Trinity College, Dublin, Ireland; 15 Barts Health NHS Trust, London, UK; 16 The University of Salford, Salford, UK; 17 Children’s Health Ireland (CHI) at Crumlin, Dublin, Ireland; 18 University of Liverpool, Liverpool, UK; 19 Maastricht University Medical Centre / Maastricht University, Maastricht, The Netherlands; 20 University Hospitals of Leicester NHS Trust and Leicester University, Leicester, UK; 21 University of Cumbria, Cumbria, UK; 22 Bangor University, Bangor, UK; 23 Paediatric RN, HSC, Lund University, Lund, Sweden; 24 Midlands Partnership NHS Foundation Trust & Keele University, Keele, UK; 25 University of Tasmania and the Royal Hobart Hospital, Tasmani, Australia; 26 University of Tasmania, Tasmania, Australia; 27 Auckland University of Technology, Auckland, New Zealand; 28 Federation University, Victoria, Australia; 29 São Paulo State University , São Paulo, Brazil; 30 Molloy College in Rockville Centre, New York City, USA; 31 Universitas Padjadjaran, Bandung Ciry, Indonesia; 32 Philadelphia University, Amman, Jordan; 33 University of Malawi, Kamuzu College of Nursing, Malawi, South Africa; 34 The Harry Crossley Children’s Nursing Development Unit, University of Cape Town, Cape Town, South Africa; 35 Dalhousie University, Halifax, Canada; 36 Ulluriaq, Ungava Tulattavik Health Center and UQAM UQO, Québec, Canada

**Keywords:** Children, Procedures, Child rights, Restraint, Consensus

## Abstract

Children continue to experience harm when undergoing clinical procedures despite increased evidence of the need to improve the provision of child-centred care. The international ISupport collaboration aimed to develop standards to outline and explain good procedural practice and the rights of children within the context of a clinical procedure. The rights-based standards for children undergoing tests, treatments, investigations, examinations and interventions were developed using an iterative, multi-phased, multi-method and multi-stakeholder consensus building approach. This consensus approach used a range of online and face to face methods across three phases to ensure ongoing engagement with multiple stakeholders. The views and perspectives of 203 children and young people, 78 parents and 418 multi-disciplinary professionals gathered over a two year period (2020–2022) informed the development of **i**nternational rights-based standards for the care of children having tests, treatments, examinations and interventions. The standards are the first to reach international multi-stakeholder consensus on definitions of supportive and restraining holds.

*Conclusion*: This is the first study of its kind which outlines international rights-based procedural care standards from multi-stakeholder perspectives. The standards offer health professionals and educators clear evidence-based tools to support discussions and practice changes to challenge prevailing assumptions about holding or restraining children and instead encourage a focus on the interests and rights of the child.

**Table:** 

**What is Known:** *• Children continue to experience short and long-term harm when undergoing clinical procedures despite increased evidence of the need to improve the provision of child-centred care.* *• Professionals report uncertainty and tensions in applying evidence-based practice to children’s procedural care.*	
**What is New:** *• This is the first study of its kind which has developed international rights-based procedural care standards from multi-stakeholder perspectives.* *• The standards are the first to reach international multi-stakeholder consensus on definitions of supportive and restraining holds.*

**Supplementary Information:**

The online version contains supplementary material available at 10.1007/s00431-023-05131-9.

## Introduction

Many children having clinical procedures (such as radiological investigations, blood tests and the administration of medicines) and interactions with healthcare professionals have positive experiences. However, many do not. There is increasing evidence of the harm which can be caused to children when they are not informed or involved in their care [1]. This harm can also occur when their expressed wishes are not listened to by adults caring for them [2, 3] or when they are held against their will to complete a procedure [4–7]. Evidence shows that the holding of children, against their will, to complete a procedure occurs frequently in practice [6], especially in younger children [8] and those with complex, sensory or additional needs [9–11]. Children who have experienced difficult procedures and health care interactions have been shown to engage less in the future with health care services including public health vaccination programs [12, 13]. They have also been shown to experience needle phobia [14], post-traumatic stress disorder (PTSD) [15] and anxiety [16]. Psychological safety is recognised as central to mental health and wellbeing and repeated exposure to trauma in health care, such as being forcefully restrained for clinical procedures can affect feelings of psychological safety and increase the risk of post-traumatic stress [17, 18]. As well as the short-and long-term impact of a poor procedural experience on a child, parents also report high levels of stress and distress [19] and a sense of eroded trust with their child [20] if they have been involved in the use of restraint during their child’s procedure.

There has been a steady increase in evidence linked to improving various elements of a child’s procedural experience and promoting a more psychologically informed approach [18, 21]. These include the provision of information and education [22–25], the use of analgesia and sedation [26, 27], the provision of distraction and guided imagery [28–30] and resources to engage children in decision-making [31, 32]. There are also evidence-based initiatives to improve children’s procedural care such as ‘Comfort Promise’ [33], ChildKind hospitals [34], CARE process [16], and Playing your CARD [35], but these are usually operationalised within specific hospitals or services. Despite the increase of evidence to shape procedural care, clinical practice continues to be inconsistent and guided by assumptions and locally accepted practices [5, 36]. Many health professionals report struggling to balance a child’s needs against pressures within health services such as shorter appointments and busy departments [24, 37]. This can result in children’s rights and their short and long-term outcomes being given less ‘weight’ than professional and institutional agendas [8]. In the midst of a procedure, professionals can lose sight of the child and experience ‘empathic blindness’ [38], raising ethical questions on procedural practices.

The continued call over the last twenty years to improve children’s procedural care by implementing evidence-based practice has led to the development of policies and guidelines to inform practice. National and international guidelines have so far focused on specific sub-sections of procedural care such as children undergoing invasive medical procedures [39], children with cancer undergoing needle procedures [40], and most recently the management of pain [41]. There was, however, no attempt to develop broad holistic guidance applicable to the wide range of procedures which children encounter every day. In addition, the existing clinical practice and policy guidelines have predominantly been developed by expert panels, neglecting the inclusion of all stakeholders involved in a child’s procedure, most notably children themselves.

The development of the rights-based standards for children having tests, treatments, examinations and interventions by the ISupport international collaboration aimed to address the need for a framework to outline and explain the rights of children within the context of a clinical procedure. The collaboration was initiated by a young person with lived experience of procedural restraint who met with the ISupport lead and called for renewed efforts to improve the procedural care of children. The ISupport international collaboration consists of multidisciplinary professionals from eighteen countries across six continents including children, young people, nurses, doctors, clinical and counselling psychologists, play specialists, patient and public involvement facilitators, radiographers, child rights specialists and parents.

## Design

The rights-based standards for children undergoing tests, treatments, investigations, examinations and interventions were developed using an iterative, multi-phased, multi-method and multi-stakeholder consensus building approach. This consensus approach used a range of methods to ensure ongoing engagement with multiple stakeholders. The approach, aligning in some respects to a modified Delphi approach [42] aimed to be inclusive in order to empower all those involved to work together to gain general agreement on the content and format of the standards. This approach is aligned to World Health Organization (WHO) [43] methods of group decision-making as a cognitive, collaborative process, whilst drawing upon multi-method multi-stakeholder consensus processes [44, 45]. The ISupport team was committed to ensuring that the standards addressed the needs and views of all those involved in children’s procedural care, including children, young people and parents from different countries. The ISupport team was formed by individual invitations sent by the lead to experts across a range of children’s health, procedural and professional networks. Team members then identified further experts within their own networks to bring additional and complementary expertise. In line with WHO guidance [43, pg204], consensus was interpreted to mean a “general acceptance by the group rather than agreement by all its members”. The multi-phased process was flexible and spanned nearly two years from January 2021 to October 2022.

## Methods

The methods used throughout the consensus building process are outlined below within three sequential phases.

### Phase one: identifying and developing the items in the standards and initial prioritisation

The first phase focussed on identifying the initial content and items to be included in the standards through decision-making meetings and consultation with youth forums.

Three rounds of decision-making meetings between the ISupport team members were held online and scheduled to be convenient to people living in different time zones. The meetings were moderated with a clear agenda and multiple parallel meetings were held with approximately 15 team members at each meeting. This aimed to facilitate discussion and meaningful contribution. Online consultation meetings were held with children and young people (see Table 1). These meetings were held with Young Person’s Advisory Groups (YPAGs) who had established relationships and working practices as advisory groups and were supported by experienced facilitators. All forums were sent materials prior to the meeting to introduce the work. The discussions were flexible and did not seek information about personal experiences but aimed to gain the views of the children and young people on what was important to include in the standards.

**Table 1:** Consultation with children, young people and parents during phase one of the consensus process

**Details of the children and young people consulted**	**Main points arising from the consultation**
Young People Hospital based Forum Ireland 11 young people aged 9–17 years 1 parent	**Content of the standards** Holding is important to focus on, but there is so much more which happens as part of a procedure. Trust is important and needs to be featured Health professionals shouldn’t get cross if you do not want to have it [procedure] done. Choice is important, children need to be asked what helps them, they are capable of being involved. Holding can help if it is supportive Agreement to a procedure can be problematic, instead of agreement it could be not being upset or resisting. **Format of the standards** There needs to be a version for children and young people There should be a sheet to take along to appointments with some of the standards on, a sheet like this would help us feel listened to. It needs to look engaging and easy to write on.
Young Person’s Advisory Group England 15 young people 10–20 years 1 parent	**Content of the standards** A sense of control is important to a good procedure. Holding makes children feel scared and out of control - it is important to describe what is good holding and what children think is ‘bad’ holding. It also matters that children have information and are listened to. The word procedure is difficult to understand and know what it means. It can be difficult to know how or when to challenge practice. **Format of the standards** The standards should be statements of ideals which every child should have e.g. Listened to, choice There should be a leaflet where parents and children can make notes and individualise their questions and information needs. This would help open up the conversation.

### Phase two: refining the items in the standards

The second phase focussed on refining the first draft of the standards through feedback from an online survey, additional consultation with youth and parent forums and decision-making meetings within the ISupport team.

An online consultation survey was sent to children, young people, parents and health professionals to gain their views on the statements developed through phase one. The survey, administered through Survey Monkey, sought anonymous feedback on the value of each of the individual items to help identify if any of the content was unclear, problematic or missing any important elements. The survey aimed to help reach consensus by using voting techniques on how ‘important’ each of the items/statements were on a three item Likert scale (very important, important, not that important). The survey listed 41 individual items for the health professional survey and 29 individual items for children, young people and parents. Open text questions aimed to encourage children, young people, parents and health professionals to provide a rationale for their rating as well as the opportunity to identify any missing content, raise issues of importance to them and query the proposed content of the items. Different versions of the survey were created for children, young people, parents and health professionals and distributed to the forums involved in phase one as well as networks known to the ISupport international team.

Two rounds of decision-making meetings were held between the ISupport team members during the second phase of the consensus building process where we discussed the feedback from the consultation surveys, the feedback from consulting with young people and parent forums as well as the evolving views and input from ISupport team members.

### Phase three: final prioritisation and reduction of items in the standards

The final phase of the consensus process involved a further online consultation survey, consultation with children and young people, two rounds of decision-making meetings between the ISupport team members and a voting process on one particular item of the standards which related to the holding and restraint of children.

Two rounds of decision-making meetings discussed the findings from the second consultation survey and the feedback from the consultation with children and young people to inform the process of consensus building.

An online survey sent to children, young people, parents and health professionals aimed to gain feedback on the revised standards and the accompanying documents developed as a result of phase two. The survey mirrored elements of the survey from phase two, seeking consensus by using voting techniques on a sliding rating scale (anchors = not good at all (0) to very good (100)) relating to either how ‘good’ or ‘important’ the revised statements were perceived to be. The survey in phase 2 was lengthy due to the inclusion of a rating scale for each individual item. For this reason, the survey in phase 3 presented all items within the seven sections (for professionals) and six sections (for children and parents) of the standards and asked for a rating on the whole section. Open text responses aimed to encourage children, parents and professionals to provide a rationale for their rating as well as providing respondents with the opportunity to identify any issues with terminology or intent. Three different versions of the survey were created for children and young people, parents and health professionals and distributed widely on professional social media channels as well as via networks known to key members of the ISupport team.

### Ethics approval

Phases one and two of the consensus process were viewed as consultation exercises involving members of youth and parent forums and the ISupport team, as such and in line with other published consensus processes and guidance from the Health Research Authority (decision-making tool), ethics approval was not required for these activities. The online survey administered as part of phase two gained ethics approval through the ISupport lead’s institution (ETH2021-0014).

During phase three of the consensus process (which involved a plan to reach out to children, young people and parents who were not part of health forums) ethical approval was required and also provided through the lead institution (ETH2021-0261). Other ISupport team members who led the recruitment of children, young people, parents and professionals within their own countries gained approvals from their employing institutions in: Australia (A21-157), Brazil (CAAE 53331321.5.0000.5411 and Opinion No 5.159.191), Spain (review was not deemed necessary by the research institute’s ethics committee as this project did not entail any direct medical intervention to children) and Sweden (Swedish Ethical Review Authority 2022-01380-01).

## Findings

### Phase one: identifying and developing the items in the standards and initial prioritisation

Three rounds of decision-making meetings with the 51 ISupport members (eight meetings in total across the time period January−March 2021) were held in phase one. These decision-making meetings enabled iterative development of the initial key items through in-depth and lengthy discussions of the scope of the work and the contrasts and similarities between procedural practice across the participating countries and contexts. In developing the standards, a rights-based approach was adopted. Such an approach ensures children’s rights, as outlined in the United Nations Convention on the Rights of the Child [46], are a key consideration in the development of policies and practices. Developing standards which reflect children’s rights within the UNCRC supports the universal application of the standards through ensuring a commonality across contexts and providing a framework to encompass country-specific laws, guidelines and regulations.

The initial items were focussed on holding and restraint but following consultation with children and young people and parents (Table 1) conducted in January and February 2021, the scope of the standards stretched to encompass the broader elements of procedural care including access to information, being listened to and the opportunity to be involved in choices and decisions. This consultation involved 26 children and young people aged 9–20 years old, from two different health forums in England and Ireland, and highlighted the importance of standards to underpin improvements in procedural care. Many of the children and young people discussed how hard it could be to share their views and have them listened to within health care services. They also highlighted how in addition to a version of the standards for professionals, there needed to be a version for children and young people so they could feel more able to share their views and choices with professionals. The two facilitators present at the forum meetings, and who were parents, shared their experiences of knowing how or when to say ‘stop’ or challenge practice within their child’s health care procedures and occasions when they felt that they should have ‘done more’ to advocate for their child.

Phase one resulted in a draft of the standards being developed.

### Phase 2: refining the items in the standards

The online consultation survey, seeking feedback on the first draft of the standards was administered between March 2021-May 2021 received 155 responses from participants in 17 countries (Australia, UK, Sweden, Canada, Malawi, Brazil, Ireland, Norway, Jordan, Netherlands, Indonesia, Zambia, South Africa, Cambodia, New Zealand, United States of America, Spain). The 110 professionals who responded worked in hospitals, community clinics, clinical commissioning groups, GP practices, voluntary organisations, training institutions and CAMHS (child and adolescent mental health services) and included doctors, nurses, play specialists, health visitors, radiographers, clinical educators, Operating Department Practitioners, youth participation workers, nursery nurses and academics. The 29 children and young people who responded were from the UK, Sweden, Spain and the Netherlands. The 26 parents and carer respondents were from the UK, the Netherlands, Cambodia and Canada. The parents endorsed the view that something to ‘take along’ to appointments would be really helpful and that a ‘preparation sheet’ would enable them to apply the standards to their child’s care. This ‘prep sheet’ which contains prompts to help families prepare and share their views during their health care interactions became part of the documents to support implementation of the standards.

A substantial proportion of health professionals, parents, children and young people rated all the items from the standards as very important on the Likert scale. The health professional ratings for each statement are included in Supplementary file 1 and the responses of children, young people and parents in Supplementary file 2. Health professionals, parents and children and young people were also asked to provide open text comments in relation to their rating and to provide additional information (Supplementary files 1 and 2), these comments were of particular value in the further development of the statements.

The open text feedback from professionals indicated that whilst the statements had merit and would impact positively on children’s procedural experiences, there were challenges within practice which were perceived as making the standards difficult to apply. The identified challenges included staff not having the necessary expertise and training to deliver child-centred preparation and information, picturing how the standards could be applied meaningfully with young children and those with learning or intellectual disabilities and how it would be too challenging to not use restraining holds as they were used frequently by staff and parents in order to deliver clinical services to children. One professional stated that prioritising children’s short AND long term interests was ‘academic and lofty’ as ‘providers are agenda driven/task-oriented due to staffing crunches and they simply need to complete procedures’.

Whilst the numeric rating scores from children, young people and parents were mainly positive, the open text comments helped the ISupport team understand the reasons for the lower ratings. The suggestions for the lower scores focussed on improving the standards for children and young people who may have specific needs, such as those who were non-verbal and may use different techniques to share their views and the way some of the language such as ‘have space’ could be interpreted literally by some young people. A few of the children and young people found the repeated use of the word ‘me’ at the beginning of each item unhelpful. Some of the statements, specifically those from parents, were more generic and highlighted the difficulties many had faced in being ‘listened to and believed by professionals’. The respondents reported that despite the content of the standards being important, they felt that health care services are lacking in time and that being kind and attending to children’s individual needs will not easily happen.

Two rounds (five meetings) of decision-making meetings with the 51 members of the ISupport team were held to consider the feedback from the survey and amend the standards accordingly. The open text survey feedback and consultation meetings were integral to the changes. Case studies were developed to demonstrate the application of the standards in practice within a range of clinical settings and with children and young people with a range of characteristics. The case studies, demonstrating the small changes which would result from the application of the standards to practice, aimed to respond to the feedback from professionals that application of the standards and reducing the use of restraining holds would be too challenging. We held further consultation meetings with sixteen young people and seven parents (Table 2). These consultations focussed around the need for the standards to be more accessible and visually appealing to children, young people and parents and for some elements of the language to be simplified and more clearly explained.

**Table 2:** Consultation with children, young people and parents during phase two of the consensus process

**Details of the children, young people and parents consulted**	**Main points arising from the consultation**
Parent group of children and young people with mental health needs 7 parents	**Content of the standards** The standards would be really relevant to children and young people within inpatient mental health services. Would they be difficult to apply in the current world we live in as many people are lacking in empathy and understanding. The NHS workers are under so much stress it might be difficult for them to deal with this. They are a really good idea. A prep sheet would help you get your point across and be heard in consultations and meetings within health services. **Format of the standards** Some of the wording, particularly the word procedure would have to change in order for it to work in a mental health setting.
Young People’s Mental Health Forum 8 young people aged 14–17 years	**Content of the standards** You would need it when going for any sort of procedure - although it might not be clear what is meant by procedure, this is not a word we are familiar with. A prep sheet should be a paper copy as well as digital to take with you - otherwise they may be like ‘turn off your phone’. I don't think a child should ever be held against their will unless the procedure is necessary and there are no other options. **Format of the standards** They are quite long to read, not many children would read them all, especially if they are unwell. They should be made as short as possible.
Young People’s Local Council Advisors 8 young people aged 16–18 years	**Content of the standards** The standards are needed as children are often ignored as they can’t talk using all the medical terms. Children and young people with learning difficulties need extra support as they can find it extra hard to make sure what matters to them is listened to. **Format of the standards** The format needs to be simplified, the length of sentences should be shortened, there should be more use of bullet points. Cannot assume that children will know what a health professional is or what against my will means. There should be some images or pictures to make it more engaging for younger children or those with additional needs. The prep sheet is useful, but additional content was identified as needed.

Phase two resulted in a revised version of the standards being developed.

### Phase 3: final prioritisation and reduction of items in the standards

Despite there being high levels of consensus (> 80%) for all items in phase two, the open text responses from health professionals, parents and children indicated that some changes to the text and items would improve the interpretation and the application and/or implementation of the standards to clinical practice.

The ISupport international team decided to conduct a further round of consultation to enable further consensus building on the set of revised items and with a wider group of children, young people, parents and professionals. We were keen to gain the views of children and young people who were not part of youth forums through a broader consultation survey and through reaching out to groups of children and young people in different contexts.

A total of 258 professionals, 28 ‘other’ adults, 43 parents and 19 children and young people responded to the survey (Table 3). The ‘other’ adults included academics working in child health, children’s charity workers, teachers, play specialists, youth workers and patient advocates. The survey collected data between August 2021-August 2022.

**Table 3:** Survey respondents in Phase 3 of the consensus building process

**Country**	**Health professional respondents** **N = 258**	**Parent respondents** **N = 43**	**‘Other’ adult respondents** **N = 28**	**Children and young people respondents** **N = 19**
UK	61	12	12	11
Australia	9	0	0	0
Spain	7	0	1	0
Sweden	51	4	0	3
Brazil	130	27	15	5

The health professionals, parents and children and young people rated all the statements from the respective version of the standards above 80 on the 0–100 sliding scale of how ‘good’ or ‘important’ overall each statement within the standards was. The ratings for each statement are presented in Tables 4 and 5. Health professionals, parents/carers and children and young people were also asked to provide open text comments in relation to their rating and to provide additional information.

**Table 4:** Phase 3 parent, children and young people ratings on the items in the standards

**Statements**	**Parent/carer rating of ‘how good’ overall the particular statements in the standards** **Score 0 (low) -100 (high)** **N = 43**	**Child or young person rating of ‘how good’ overall the particular statements in the standards** **Score 0 (low)-100 (high)** **N = 19**
**Communicating with me**	81/100	100/100
**Making choices and decisions with me**	82/100	70/100
**Sharing information with me and helping me prepare**	87/100	93/100
**Acting in a way where my well-being comes first**	87/100	89/100
**Holding me**	84/100	100/100
**Documenting my procedure**	91/100	89/100

**Table 5:** Phase 3 professional ratings on importance of the items in phase 3 of the consensus process

**Statements**	**Rating of ‘how good’ overall the particular statements in the standards** **Score 0 (low) -100 (high)** **N = 258**
A child has rights to be cared for by professionals who have the appropriate knowledge and skills to support their physical, emotional and psychological well-being and rights before, during and after their procedure	92/100
A child has rights to be communicated with in a way which supports them to express (verbally or behaviourally) their views and for these views to be listened to, taken seriously and acted upon	93/100
A child has rights to be provided with meaningful, individualised and easy to understand information to help them prepare and develop skills to help them cope with their procedure	91/100
A child has rights to be supported to make procedural choices and decisions and for these choices to be acted upon to help them gain some control over their procedure	91/100
A child has the right for their short and long term best interests and well-being to be a priority in all procedural decisions	92/100
A child has the right to be positioned for a procedure in a supportive hold (if needed) and should not be held against their will	88/100
A child’s health records should include clear documentation of a procedure and any use of restraining holds	91/100

The ratings on the individual statements from children, young people and parents were all above 80, apart from the children and young people’s rating of making choices and decisions (Table 4, with full details in Supplementary file 3). Children, young people and parents' open text comments were focussed on how important the standards were to prevent trauma from procedures and improve experiences. Similar to the findings of the consultation survey in Phase 2, several parents acknowledged how difficult it will be to implement the standards in practice due to professionals and services being ‘too busy’ to spend time with children. Many comments highlighted the need to simplify some of the text to make it more accessible to families.

Health professional ratings of each individual statement within the standards were all above 90% (Table 5, full details in supplementary file 4), apart from the items relating to holding (88). The open text feedback from professionals was focussed on the need to reconsider the use of developmental language in the statements and the challenges which would be faced within constrained and busy services to apply the standards in practice. Of particular interest were the many statements from professionals indicating that conducting procedures without the use of restraint for the many procedures which are not an emergency or lifesaving would be ‘problematic’ and ‘challenging’ and would limit their ability to carry out their work. Feedback indicated the need to extend the remit of the use of restraining holds to procedures where significant harm would be caused if a procedure was not completed. Professionals also responded to highlight that the addition of a ‘dental’ case study and one featuring an older child within a mental health setting would be useful to include.

One round (two meetings) of decision-making meetings were held to discuss the feedback at length. As outlined above, whilst the ratings of the items were high, the comments within the open text responses required the team to discuss the revised statements. There were extended conversations to further refine the definition of supportive holding and restraining holds, alterations were made by removing the word ‘should’ within each statement as this was seen to imply that the items were optional and the feedback led to some of the words in the items being altered. At this stage two of the main statements were condensed into one leading to six overall statements in the professional version and five statements in the family version of the standards. The final phase involved an anonymous online voting exercise conducted by 36 members of the ISupport international collaboration to make and agree a final boundary for the use of a restraining hold in October 2022 (Table 6).

**Table 6:** Final decision-making voting for agreement of the boundary to the use of a restraining hold

**Suggested definition of the use of restraining holds**	**ISupport team rating** **N = 36** **% (n)**
A child should not be held against their will (restrained) at anypoint in a procedure unless the procedure is life saving or an emergency	25% (9)
A child should not be held against their will (restrained) at any point in a procedure unless the procedure is life saving, an emergency or is part of a carefully documented and agreed pre-procedure multidisciplinary plan.	19% (7)
A child should not be held against their will (restrained) at any point in a procedure unless the procedure is life saving, an emergency or where there is a likelihood of significant harm if the procedure is not carried out.	42% (15)
Other	14% (5)

The consultation with children and young people in school and activity settings in phase 3 helped to improve the final content and format of the standards, addressing children’s calls throughout the process for more colour, simpler language and the inclusion of pictures (Table 7).

**Table 7:** Consultation with children, young people and parents during phase three of the consensus process

**Details of the children and young people consulted**	**Main points arising from the consultation**
Young People’s International Rights Conference 87 young people, from 23 countries and 4 continents aged 12–23	**Content of the standards** The standards are important, adults still think children’s opinions are not important, they think we are too young to understand. It is important to focus on rights as if children are not informed of their rights then they can’t have them. **Format of the standards** The sections are clear and important, but the wording needs to be ‘made more simple’ for children.
Young People with Special Educational Needs and Disabilities attending a social activity club 8 young people aged 14–18 years	**Content of the standards** These are all important things. It is really difficult to say stuff when you are in the hospital, they do not listen to you. If holding is by someone you trust it can help. **Format of the standards** There needs to be pictures, like the ones we use in school. There is too much writing.
Children from a ‘rights’ group within a primary school setting 18 children aged 9–10 years	**Content of the standards** The standards are good, it is important for children to have rights. **Format of the standards** The faces need to be smiley not blank as they look creepy otherwise The pictures used in the international rights are good - they apply to all kinds of children. It is hard to get one picture to say ‘all that’ [one section].

The 26 children and young people aged 9–18 years from the school and social activity club settings were asked to draw what image or ‘icon’ could accompany each of the sections in the standards by free drawing what they thought of when each section was mentioned and how we could ‘show’ the key ideas in images. The children and young people in the school (n = 18) were then asked to ‘vote’ (give a mark out of 10) for each style from a range of six illustration styles used in previous resources. These were then tallied and used to underpin the family version of the standards.

**Fig. 1:**
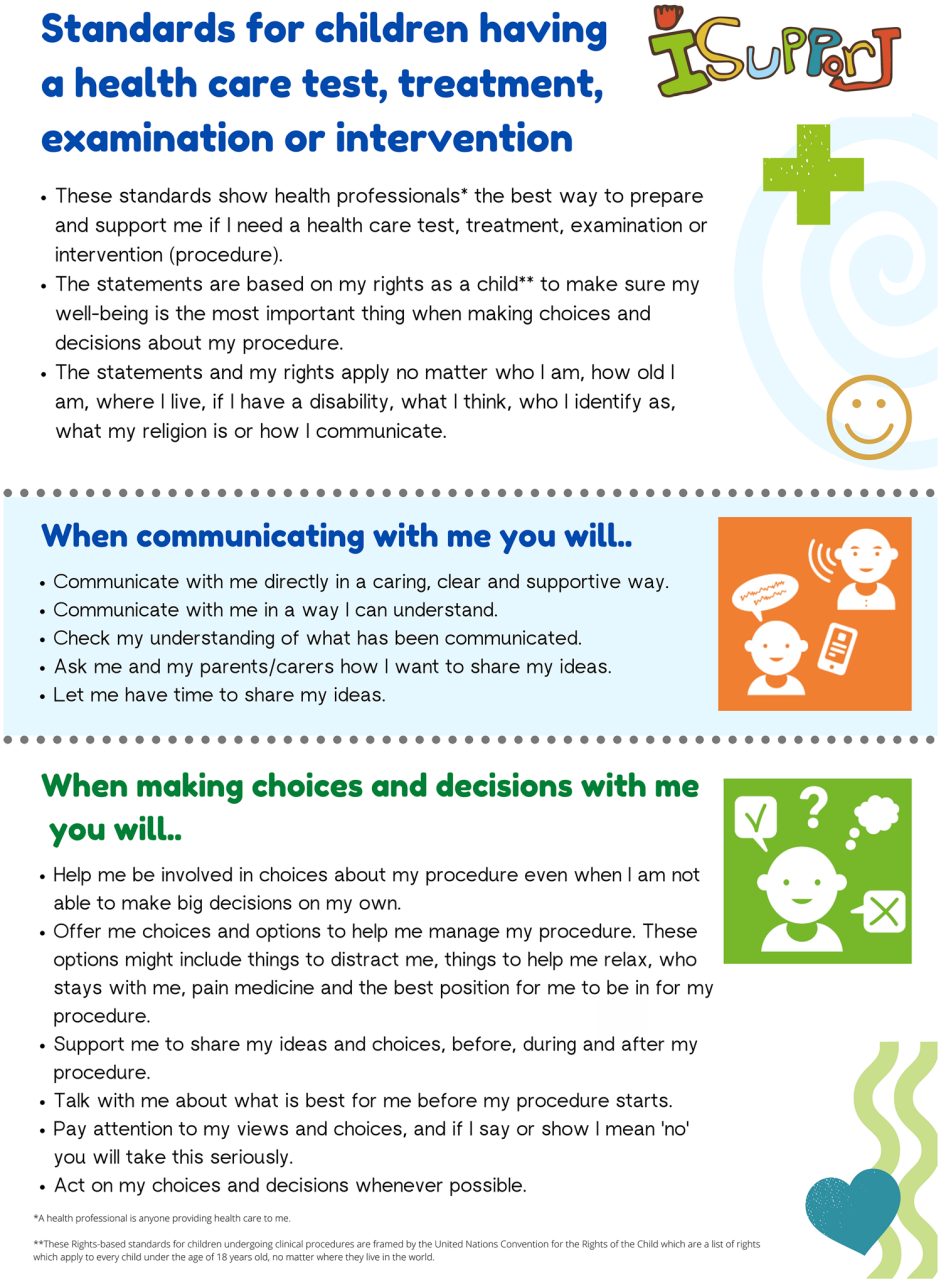
Standards for families

**Fig. 2:**
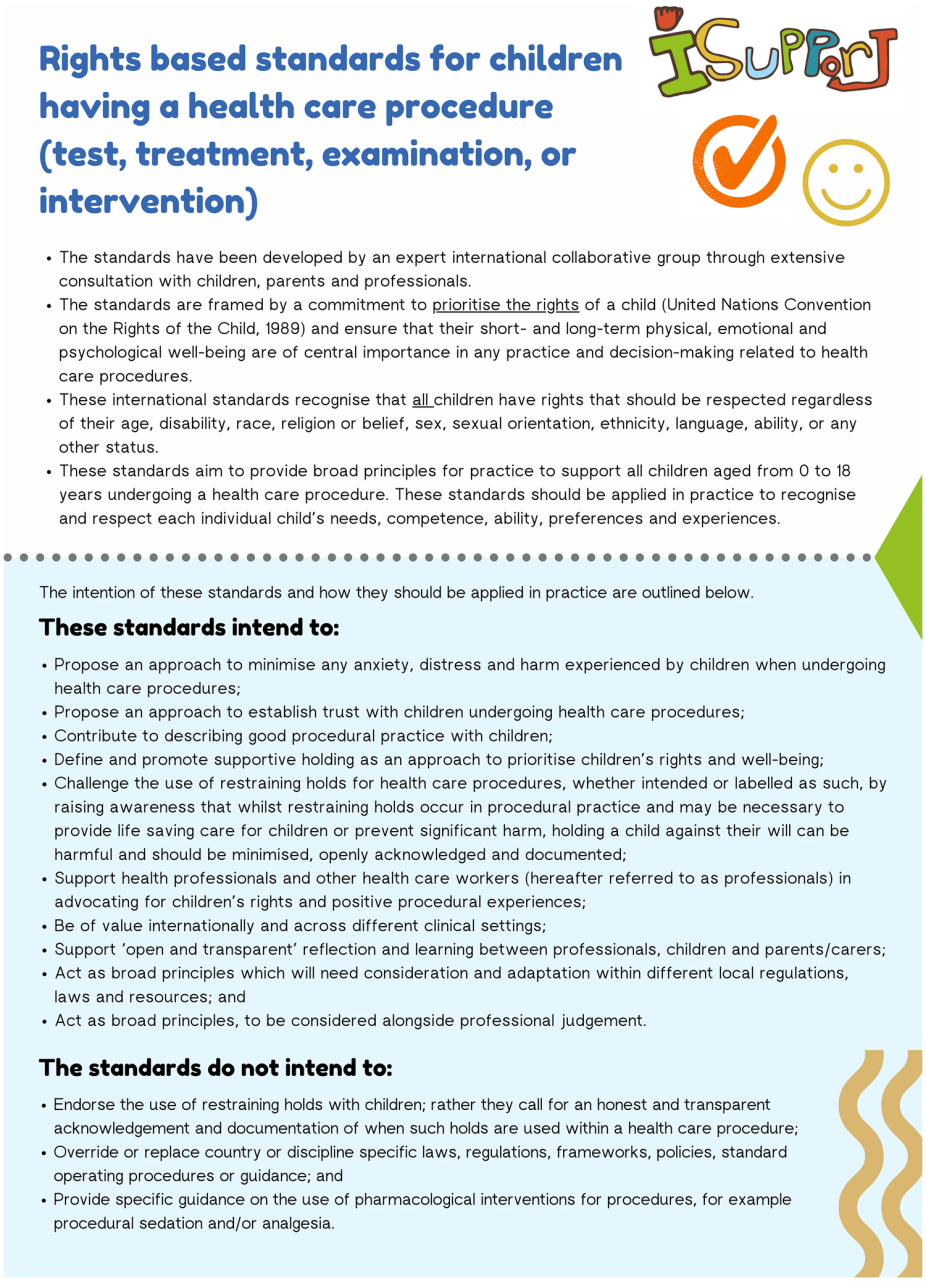
Standards for professionals

The final version of the rights-based standards for children having tests, treatments, examinations and interventions were completed in October 2022. The standards and accompanying documents are free to download and use and are shown in Figs. 1 and 2. The accompanying documents involve a version of the standards for professionals, one for children, young people and families, a preparation sheet, case studies to demonstrate the application of the standards to practice and a table of evidence. The standards have been translated to Spanish, Brazilian Portuguese, Dutch and Swedish through a translation and back translation process. This involved carefully examining and making decisions on terminology to ensure the translated versions retained the core elements and terms used in the original English language version.

Minor amendments were made to the icon images after further consultation with 108 children aged 7–17 years with Special Education Needs and Disabilities at a youth conference in March 2023 in the UK.

### Strengths and limitations

The inclusion of children, young people, parents and multi-disciplinary professionals from international countries was a strength of the project as was the lengthy iterative process which underpinned the consensus process. The use of multiple methods to enable children, young people, parents and professionals to share their views ensured that multiple perspectives were incorporated in shaping the standards. A limitation of the work is that the process engaged with a convenience and snowball sample of stakeholders and could have included further engagement from children, young people, parents and professionals from Africa and Asia.

## Conclusion

Through a phased, multi-method, multi-stakeholder consensus building process, rights-based standards were developed for children having tests, treatments, examinations and interventions. It is believed that this is the first study of its kind which outlines international rights-based procedural care standards from multi-stakeholder perspectives. The standards are the first to reach international multi-stakeholder consensus on definitions of supportive and restraining holds. It is hoped that the standards will be an important step in improving the procedural care of children and young people especially given that they are grounded in a framework of children's rights, regardless of jurisdiction, thus giving them universality. The standards offer health professionals and educators clear evidence-based tools to support discussions and practice changes to challenge prevailing assumptions about holding or restraining children and instead encourage a focus on the interests and rights of the child. The implementation of the standards into practice will require a sustained approach to engage professionals, policy makers, children and parents.

### Implications for practice and research

- The practice of holding children for procedures is often shrouded in uncertainty and confusion, these standards are the first to clearly define supportive and restraining holds. It is hoped that this will enable professionals to acknowledge and document when practice has aligned with restraint and therefore begin to improve procedural practice and the support offered to children.
- The creation of a version of the standards for professionals and one for families aims to encourage open and collaborative procedural expectations to be established and met.
- These international standards hope to enable clear auditing of procedural practice.
- The international standards are free to access and download at https://www.isupportchildrensrights.com/.

### Supplementary Information

Below is the link to the electronic supplementary material.

Supplementary file1 (DOCX 24 KB)

Supplementary file2 (DOCX 22 KB)

Supplementary file3 (DOCX 20 KB)

Supplementary file4 (DOCX 17 KB)

## Data Availability

Data are available on request due to ethical restrictions.
