# Supplementary material for: Developing rights-based standards for children having tests, treatments, examinations and interventions: using a collaborative, multi-phased, multi-method and multi-stakeholder approach to build consensus
Source: Eur J Pediatr. 2023 Aug 11;182(10):4707–21. doi: 10.1007/s00431-023-05131-9 (PMC10587267; doi:10.1007/s00431-023-05131-9)
Supplement: Supplementary file 1 — Supplementary file1 (DOCX 24 KB) [file 431_2023_5131_MOESM1_ESM.docx]

Supplementary File 1; Health professional ratings of items in Phase 2 of consensus building process

|  | **Survey ratings for professionals and others (N=110** **)  %  (n)** | | | |
| --- | --- | --- | --- | --- |
|  | Very important | Not that important | Not important at all | Needs to change |
| **A child has rights to be cared for by professionals appropriately trained and educated to support their physical, emotional and psychological well-being and rights before, during and after their procedure.** | | | | |
| A child is cared for by a health professional who has the appropriate knowledge and skills and who is  competent to conduct the clinical procedure. | 98% (108) | 1% (1) | 0  (0) | 1% (1) |
| A child is cared for by a health professional who has access to the right equipment and other resources (e.g., staff, environment) to conduct the procedure | 96% (106) | 1% (1) | 0 (0) | 3% (3) |
| A child is cared for by a health professional who has ascertained and confirmed the clinical need and requirement of the procedure. | 96% (105) | 4% (4) | 0 (0) | 1% (1) |
| A child is cared for by a health professional who has the appropriate knowledge and skills to assess a child’s developmental level and abilities. | 95% (104) | 5% (6) | 0 (0) | 0 (0) |
| A child is cared for by a health professional with knowledge of, and respect for children’s rights and how to advocate for children’s rights. | 96% (105) | 3% (3) | 0  (0) | 2% (2) |
| A child is cared for by a health professional who has the appropriate knowledge and skills to ensure procedural comfort and to reduce the potential for traumatic procedural experiences | 99% (109) | 1% (1) | 0  (0) | 1% (1) |
| **A child has rights to be communicated with in a way which supports them to express their views and for these views to be listened to, taken seriously and acted upon** | | | | |
| A child is communicated with directly in an open, honest, warm, supportive and caring way to appropriately acknowledge their feelings, and in a way that they can understand and that is consistent with their development at the time of the procedure. | 97% (107) | 1% (1) | 0 (0) | 2%  (2) |
| A child is provided with the space, time and environment to feel able to communicate and express their views and develop trust and rapport with those present at their procedure. | 95% (105) | 3% (3) | 0 (0) | 3% (0) |
| A child is encouraged to voice their opinions freely without pressure, coercion or manipulation. | 99% (108) | 0  (0) | 0 (0) | 1% (1) |
| A child is supported to recognise, communicate and action their rights. | 95% (105) | 3% (3) | 0 (0) | 2%  (2) |
| A child’s parents/carers are provided with support to recognise and communicate their child’s rights. | 97% (107) | 2%  (2) | 0 (0) | 2% (2) |
| A child should be praised for any part of a procedure they have managed. | 95% (104) | 4% (4) | 0 (0) | 3% (3) |

| **A child has rights to be supported to make procedural choices and decisions and for these choices to be acted upon to help them gain some control over their procedure.** | | | | |
| --- | --- | --- | --- | --- |
| A child should be assumed to have the ability to be involved in decisions and choices about their procedure even when they are not able to make decisions on their own. | 89%     (97) | 6%   (6) | 1%       (1) | 5%       (5) |
| A child should be provided with sufficient information to enable them to form their own views and be involved in decisions and choices about their procedure | 92% (101) | 4% (4) | 0  (0) | 5% (5) |
| A child should be informed of the consequences of decisions and choices about their procedure in a non-biased way | 91% (99) | 5% (5) | 0  (0) | 6% (6) |
| A child should be actively encouraged from the earliest opportunity and throughout the procedure to share their procedural views, preferences and choices. This may include analgesia, methods of distraction, relaxation techniques, who supports them for their procedure and sources of comfort. | 95% (103) | 4% (4) | 0 (0) | 2% (2) |
| A child should be supported through their choices and decisions to gain control over their procedure. | 94% (101) | 1% (1) | 1% (1) | 1% (1) |
| A child and their parent/carer should be provided with the opportunity to discuss previous procedural experiences to inform procedural choices and decisions. | 94%  (102) | 7% (8) | 0 (0) | 0 (0) |
| A child’s parent/carers are supported by health professionals who will work with them to apply the child’s choice and preference for non-pharmacological techniques. | 92% (100) | 4% (4) | 0  (0) | 6% (6) |
| A child’s views, choices and expressions of dissent should be listened to, considered and taken seriously and given due weight in accordance with the age and maturity of the child. | 94% (101) | 4% (4) | 1% (1) | 3% (3) |
| **A child has rights to be provided with meaningful and individualised information to help them prepare and develop skills to help them cope with their procedure.** | | | | |
| A child should be provided with easy to understand, meaningful and honest non-biased information to ensure they are aware and prepared for a procedure, understand what is happening, and has the opportunity to ask questions to check their understanding. | 97% (106) | 1% (1) | 0 (0) | 4% (4) |
| A child’s parent/carer should be provided with easy to understand, meaningful and honest information to ensure they are aware and prepared for their child’s procedure, understand what is happening, and has had the opportunity to ask questions and be actively involved in their child’s care. | 98% (106) | 1% (1) | 0 (0) | 1% (1) |
| A child should receive information that is appropriately timed and individually tailored to their needs, developmental stage, cognitive level and abilities. | 98% (107) | 1%  (1) | 0 (0) | 1% (1) |
| A child should be involved in any plans for their procedure | 82% (89) | 12% (13) | 1% (1) | 6% (6) |
| A child’s questions and expressions of concern should be responded to in a calm, honest and developmentally appropriate manner. | 100% (108) | 0% (0) | 0% (0) | 0% (0) |
| A child’s information needs should be continually checked at key points throughout their procedure. | 92% (99) | 6% (7) | 1% (1) | 2% (2) |
| **A child has the right for their short and long term best interests and well-being to be a priority in all procedural decisions** | | | | |
| A child’s best interests are the top priority in all decisions and actions before, during and after a clinical procedure. A child’s best interests should be prioritised over those of their parents, health professionals and the institution | 93% (100) | 3% (3) | 1% (1) | 6% (6) |
| A child’s short and long-term best interests and developmental level should be openly considered and collectively discussed by health professionals, parents and the child (where appropriate) in the preparation phase prior to the procedure | 98% (106) | 2% (2) | 0% (0) | 0% (0) |
| A child should be protected from harm; any potential or actual harm to a child caused by unnecessary procedures or overriding their expressions of dissent needs to be carefully considered and mitigated wherever possible. | 100% (107) | 0% (0) | 0% (0) | 0% (0) |
| A child’s procedure has an identified health professional ‘lead’ who will be responsible for advocating for a child’s best interests and engage using a ‘one voice’ approach during the procedure. | 87% (93) | 9% (10) | 1% (1) | 8% (9) |
| A child should be supported to remain calm, secure and settled during a procedure. | 97% (104) | 3% (3) | 0% (0) | 1%  (1) |
| A child who becomes upset or resistant before or during a procedure should be facilitated/ helped as quickly as possible to take a supported break and for health professionals  to feel confident to stop and reconsider the procedural plan. | 94% (100) | 4% (4) | 0% (0) | 6% (6) |
| A child is supported after a procedure to understand and frame or re-frame their experience. | 95%  (100) | 5% (5) | 0% (0) | 0%  (0) |
| **A child has the right to be positioned for a procedure in a supportive hold (if needed) and should not be held against their will.** | | | | |
| A child should only be held using a supportive hold for their procedure and should not be held against their will (restrained) at any point in a procedure unless the procedure is a clinical emergency to sustain life or prevent a child from harming themselves or others. | 92% (99) | 3% (3) | 0%  (0) | 9%  (10) |
| A child should be encouraged to express their views and choices for who will supportively hold them for their procedure. | 95%  (103) | 4%  (4) | 0% (0) | 1%  (1) |
| A child should actively agree to a procedure and being supportively held; this agreement cannot occur if the child expresses signs of resistance (verbally and/or behaviourally) to the procedure or supportive hold. | 80% (86) | 8% (9) | 3%   (3) | 10%  (11) |
| A **supportive hold** involves supporting a child to remain calm, secure and settled during a procedure. In a supportive hold a child agrees to the procedure and positioning or does not express signs of resistance. Supportive holding is a way of providing comfort to the child and helping them to maintain a good position for the procedure. | 95% (103) | 1%  (1) | 0% (0) | 4% (4) |
| A **restraining hold**, is any action to prevent a child moving freely against their choice or will. Regardless of who holds a child, if it is against their choice or will (expressed verbally and/or behaviourally) the hold is a restraining hold. A restraining hold should be recognised as such and not labelled as a clinical, supportive or comfort hold. | 88% (95) | 1%  (1) | 1%  (1) | 9%  (10) |
| A child who has experienced a restraining hold during a procedure should have this considered as an untoward incident (sometimes called a ‘red flag incident’) and this should be appropriately reported. This will enable an appropriate investigation to take place about *why* a child was subject to a restraining hold and to produce recommendations of how this could be avoided in the future | 82%  (88) | 7%   (7) | 0%  (0) | 12% (13) |
| Any child or young person who has been subjected to a restraining hold during a procedure should receive appropriate support from a health professional to help them re-frame their experience and re-build trust. | 99%  (106) | 0%  (0) | 0% (0) | 1% (1) |
| **Children’s medical records should document procedural choices and any use of restraining holds** | | | | |
| A child’s medical records will include clear documentation if they have been held without their agreement (restraining hold), regardless of who held the child. This would include the rationale for using a restraining hold, who made the decision that a restraining hold was necessary, the restraining hold/technique(s) used, and the outcome for the child or young person. This documentation aims to support ‘open and transparent’ reflection and learning, hand-over between departments and professionals and result in recommendations for a child’s future procedures. | 91% (98) | 5% (5) | 0%  (0) | 6%  (7) |
| A child’s medical records should include clear documentation of what worked well during a procedure and what procedural support or techniques would help for future procedures. | 95% (103) | 5% (5) | 0%  (0) | 1%  (1) |
