# Supplementary material for: Developing rights-based standards for children having tests, treatments, examinations and interventions: using a collaborative, multi-phased, multi-method and multi-stakeholder approach to build consensus
Source: Eur J Pediatr. 2023 Aug 11;182(10):4707–21. doi: 10.1007/s00431-023-05131-9 (PMC10587267; doi:10.1007/s00431-023-05131-9)
Supplement: Supplementary file 2 — Supplementary file2 (DOCX 22 KB) [file 431_2023_5131_MOESM2_ESM.docx]

Supplementary file 2; Child and Parent ratings of the items in the standards in phase two of the consensus process.

|  | **Child/young person ratings**  **N=19 % (n)** | | | | **Parent/carer ratings**  **N=26 % (n)** | | | |
| --- | --- | --- | --- | --- | --- | --- | --- | --- |
|  | Very important | Not that important | Not at all important | Needs to change | Very important | Not that important | Not at all important | Needs to change |
| **Communicating with me** | | | | | | | | |
| You will communicate with me directly in a caring, clear and supportive way. | 95%  (18) | 5%  (1) | 0 | 0 | 100% (26) | 0 | 0 | 0 |
| You will communicate with me in a way I can understand. | 95% (18) | 5% (1) | 0 | 0 | 100% (26) | 0 | 0 | 4% (1) |
| You will ask me or my parents/carers how I want to communicate my ideas. | 84% (16) | 16% (3) | 0 | 5%  (1) | 86% (23) | 12% (3) | 0 | 4% (1) |
| You will help my parents/carers to support my rights and let me have space to share my ideas. | 84% (16) | 16% (3) | 0 | 5% (1) | 96% (25) | 4% (1) | 0 | 8% (2) |
| **Making choices and decisions with me** | | | | | | | | |
| You will accept that I can be involved in decisions and choices about my procedure even when I am not able to make decisions on my own | 84%   (16) | 5%  (1) | 0 | 11% (2) | 96% (25) | 4% (1) | 0 | 0 |
| You will actively support me before, during and after my procedure to share my ideas and choices | 89%  (17) | 5%  (1) | 0 | 5% (1) | 96% (25) | 4% (1) | 0 | 0 |
| You will offer me choices and options to help me get through my procedure; these options might include things to distract me, things to help me relax, sources of comfort, who stays with me, pain medicine and the best position for me to be in for my procedure. | 95% (18) | 5% (1) | 0 | 0 | 100% (26) | 0 | 0 | 4% (1) |
| You will listen to my views, choices and expressions of refusal and take them seriously. | 100%  (19) | 5% (1) | 0 | 0 | 96% (25) | 4% (1) | 0 | 0 |
| You will act on my choices and decisions whenever possible. | 89%  (17) | 5%  (1) | 0 | 0 | 100% (26) | 5% (1) | 0 | 0 |
| **Sharing information with me and helping me prepare** | | | | | | | | |
| You will give me easy to understand and honest information to make sure I am prepared for my procedure. | 89% (17) | 11% (2) | 0 | 0 | 96% (25) | 0 | 0 | 8% (2) |
| You will help me understand what is happening and give me chances to ask questions. | 100% (19) | 0 | 0 | 0 | 100%  (25) | 0 | 0 | 0 |
| You will give my parents/carers easy to understand and honest information to ensure they are aware and prepared for my procedure, understand what is happening, and have the chance to ask questions. | 95% (18) | 5%  (1) | 0 | 0 | 100% (26) | 0 | 0 | 0 |
| **Acting where my well-being comes first** | | | | | | | | |
| You will think about what is best for me in all decisions and actions before, during and after my procedure. | 89% (17) | 11% (2) | 0 | 0 | 96% (25) | 0 | 0 | 4% (1) |
| What is best for me should come first before what is best for my parents, the health professionals and the hospital or clinic. | 74% (14) | 26%  (5) | 0 | 0 | 100% (26) | 0 | 0 | 0 |
| You will talk with me about what is best for me before my procedure starts. | 84% (16) | 16% (3) | 0 | 0 | 85% (22) | 8% (2) | 0 | 0 |
| You need to consider how it will make me feel if you do not listen when I say or show I mean ‘stop’ or ‘no’. | 95% (18) | 5%  (1) | 0 | 0 | 100% (26) | 0 | 0 | 0 |
| You will make sure I can choose  one person who will guide me through my procedure and make sure what is best for me happens. | 74% (14) | 26%  (5) | 0 | 0 | 96% (25) | 4%  (1) | 0 | 0 |
| You will help me to remain calm and feel listened to during my procedure and should stop (if it is safe to do so) if I say or show I mean ‘stop’ or ‘no’. | 95% (18) | 5% (1) | 0 | 0 | 96% (25) | 4% (1) | 0 | 4% (1) |
| You will make sure I actively agree to a procedure and a supportive hold and I do not show resistance (verbally or non-verbally) to the procedure or a supportive hold. | 84% (16) | 16%  (3) | 0 | 0 | 81% (21) | 4% (1) | 0 | 0 |
| You will support me to take a break if I become upset during a procedure to help everyone re-think how to help me have my procedure. | 84% (16) | 16% (3) | 0 | 0 | 96%  (24) | 4% (1) | 0 | 0 |
| Re-thinking things to help me might involve trying again another time, involving a play specialist or using sedation. | 84% (16) | 16% (3) |  | 5% (1)      0 | 100% (26) | 0 | 0 | 0 |
| The health care professional will not hold me against my will or expect my parent or carer to hold me against my will to get a procedure done. | 79% (15) | 16%  (3) | 5% (1) | 0 | 88% (22) | 8% (2) | 0 | 0 |
| You will support me after my procedure to help me understand my experience. If I have been held against my wishes you should offer me follow-up support from a health professional. | 84%  (16) | 16% (3) | 0 | 0 | 92% (23) | 4% (1) | 0 | 8% (2) |
| **Holding me** | | | | | | | | |
| If you are holding me, this must be supportive holding. Supportive holding helps me keep still and feel calm, safe and settled during a procedure. If a hold is supportive I will have agreed to being held. | 95% (18) | 5% (1) |  | 5%  (1)  0 | 92% (24) | 4% (1) | 0 | 12% (3) |
| You will ask me how and who I would like to hold me. | 95% (18) | 5% (1) | 0 | 0 | 96% (25) | 4% (1) | 0 | 4% (1) |
| If someone (no matter who that is) is holding me against my will then it is a restraining hold. | 64%  (11) | 25% (4) | 0 | 19%  (3) | 92% (24) | 4% (1) | 0 | 8%  (2) |
| You will not hold me against my will unless my procedure is an emergency or if you are worried I will hurt myself or another person | 95% (18) | 5%  (1) | 0 | 0 | 88% (23) | 4%  (1) | 0 | 15%  (4) |
| **Documenting my procedure** | | | | | | | | |
| You will record key points about the procedure and any holding used  in my notes and what helped me/didn’t help me as this will be useful for any future procedures. | 84% (16) | 11% (2) | 5% (1) | 0 | 88% (23) | 8% (2) | 0 | 8% (2) |
