# Supplementary material for: Developing rights-based standards for children having tests, treatments, examinations and interventions: using a collaborative, multi-phased, multi-method and multi-stakeholder approach to build consensus
Source: Eur J Pediatr. 2023 Aug 11;182(10):4707–21. doi: 10.1007/s00431-023-05131-9 (PMC10587267; doi:10.1007/s00431-023-05131-9)
Supplement: Supplementary file 4 — Supplementary file4 (DOCX 17 KB) [file 431_2023_5131_MOESM4_ESM.docx]

Supplementary file 4;  Phase 3 professional ratings on importance of all the items in phase 3 of the consensus process

| **Statements** | **Rating of ‘how good’ overall the particular statements in the standards**  **0-100**  **N=258** |
| --- | --- |
| **A child has rights to be cared for by professionals who have the appropriate knowledge and skills to support their physical, emotional and psychological well-being and rights before, during and after their procedure**  a) A child is cared for by a professional who has the appropriate knowledge and skills and who is competent to conduct the clinical  procedure.  b) A child is cared for by a professional who has access to appropriate equipment and resources (e.g. staff, environment) to conduct the  procedure.  c) A child is cared for by a professional who has confirmed the clinical need for the procedure.  d) A child is cared for by a professional who has the appropriate knowledge and skills to assess a child’s individual developmental level  and abilities.  e) A child is cared for by a professional who demonstrates respect for children’s rights and who can work in a child-centred manner to  support and advocate for these rights.  f) A child is cared for by a professional who has the appropriate knowledge and skills to promote procedural comfort and to reduce the  potential for traumatic procedural experiences.  g) A child is cared for by a professional who can work in partnership with a child and their parents/carers and who can utilise the skills  and knowledge of the wider multidisciplinary team (if available) | 92/100 |
| **A child has rights to be communicated with in a way which supports them to express (verbally or behaviourally) their views and for these views to be listened to, taken seriously and acted upon.**  a) A child is communicated with directly in an open, honest, supportive and caring way to appropriately acknowledge their feelings and  in a way a child can understand and that is consistent with their development at the time of the procedure.  b) A child is provided with the space, time and environment to develop trust and rapport with those present at their procedure.  c) A child is provided with the space, time and environment to feel able to communicate and freely express their views and feelings  before, during and after their procedure.  d) A child is encouraged and supported to express their views and feelings freely without pressure, coercion or manipulation.  e) A child is encouraged and supported to recognise and communicate their rights.  f) A child’s parents/carers are supported to recognise and communicate their child’s views, choices and rights. | 93/100 |
| **A child has rights to be provided with meaningful, individualised and easy to understand information to help them prepare and develop skills to help them cope with their procedure.**  a) A child should be provided with tailored, easy to understand, meaningful and honest information to ensure their preferences are taken into account, and that they are aware and prepared for a procedure, understand what is happening and have the opportunity to ask questions to check their understanding.  b) A child should receive specific, honest and clear information at key points before, during and after their procedure.  c) A child’s questions and expressions of concern should be responded to in a calm, honest and developmentally appropriate manner.  d) A child’s parents/carers should be provided with tailored, appropriately timed, easy to understand, meaningful and honest information to ensure they are aware and prepared for their child’s procedure and have been able to ask questions to understand what is happening and their role in supporting their child before, during and after a procedure. | 91/100 |
| **A child has rights to be supported to make procedural choices and decisions and for these choices to be acted upon to help them gain some control over their procedure.**  a) A child should be assumed to have the ability to be involved in choices about their procedure even when they are not able to make bigger decisions on their own.  b) A child should be provided with sufficient information, including alternate options and outcomes, in ways that enable them to form their own views and be involved in choices and decisions about their procedure.  c) A child should be actively encouraged from the earliest opportunity and throughout the procedure to share their views, procedural preferences and choices. This may include analgesia, methods of distraction, relaxation techniques, positioning, who supports them for their procedure and sources of comfort.  d) A child should be supported through their choices and decisions to have some control during their procedure.  e) A child and their parents/carers should be provided with the opportunity to discuss previous procedural experiences to inform procedural choices and decisions.  f) A child’s parents/carers should be supported by a professional who works with them to consider their child’s views, preferences and procedural choices for pharmacological and non-pharmacological techniques.  g) A child’s views, procedural choices and expressions of refusal should be listened to, considered and taken seriously and given due weight. | 91/100 |
| **A child has the right for their short and long term best interests and well-being to be a priority in all procedural decisions**  a) A child’s best interests are prioritised in all decisions and actions before, during and after a procedure. A child’s interests should be prioritised over those of their parents, professionals and the institution.  b) A child’s short and long-term best interests should be openly considered and collectively discussed by health professionals, parents and the child (where appropriate) in the preparation phase prior to the procedure.  c) A child should be protected from harm; any potential or actual harm to a child caused by unnecessary procedures or overriding their expressions of dissent should be carefully considered and mitigated wherever possible.  d) A child should be supported to feel calm, secure and settled during a procedure.  e) A child who becomes upset or resistant before or during a procedure should be helped as quickly as possible, if it does not cause harm, to take a supported break. Professionals should be confident to stop and reconsider the procedural plan.  f) A child and their parents/carers are supported after a procedure to talk through their experiences and reflect on positive or any challenging aspects. | 92/100 |
| **A child has the right to be positioned for a procedure in a supportive hold (if needed) and should not be held against their will.**  a) A supportive hold involves supporting a child to feel calm, secure and settled during a procedure. In a supportive hold a child agrees to the procedure and positioning and/or does not express signs of refusal. Supportive holding is a way of providing comfort to the child and helping them to maintain a good position for the procedure.  b) A restraining hold is any action to prevent a child moving freely against their choice or will. Regardless of who holds a child, if it is against their will (expressed verbally and/or behaviourally) the hold is a restraining hold. A restraining hold should be recognised as such and not labelled as a clinical, supportive or comfort hold.  c) A child should only be held using a supportive hold for their procedure and should not be held against their will (restrained) at any point in a procedure unless the procedure is life saving or an emergency or prevent a child from harming themselves or others.  d) A child should be encouraged to express their views and choices about who will supportively hold them for their procedure.  e) Any child who has been subjected to a restraining hold during a procedure should receive appropriate support from a professional to help them talk through and understand their experience and re-build trust. | 88/100 |
| **A child’s health records should include clear documentation of a procedure and any use of restraining holds**  a) A child’s health records should include clear documentation of what worked well during a procedure and what procedural support or techniques would help for future procedures.  b) A child’s health records will include clear documentation if they have been held without their agreement (restraining hold), regardless of who held the child. This would include the rationale for using a restraining hold, who made the decision that a restraining hold was  necessary, the restraining hold/technique(s) used, and the outcome for the child or young person. This documentation aims to support ‘open and transparent’ reflection and learning, hand-over between departments and professionals and result in recommendations for a child’s future procedures. | 91/100 |
